# Scleredema of Buschke leading to an ulcerated plaque on the neck

**DOI:** 10.1002/ski2.311

**Published:** 2023-12-05

**Authors:** Thu M. Truong, Timothy Makkar, Amy Pappert

**Affiliations:** ^1^ Center for Dermatology Rutgers Robert Wood Johnson Medical School Piscataway New Jersey USA; ^2^ Department of Pathology and Laboratory Medicine New Jersey Medical School Newark New Jersey USA

## Abstract

Scleredema diabeticorum (SD) is a common cause of scleredema with limited effective treatment options available. Patients with SD may experience significant discomfort due to symptoms of itching, burning, or pain. SD typically develops due to poor glycaemic control as demonstrated in this case.

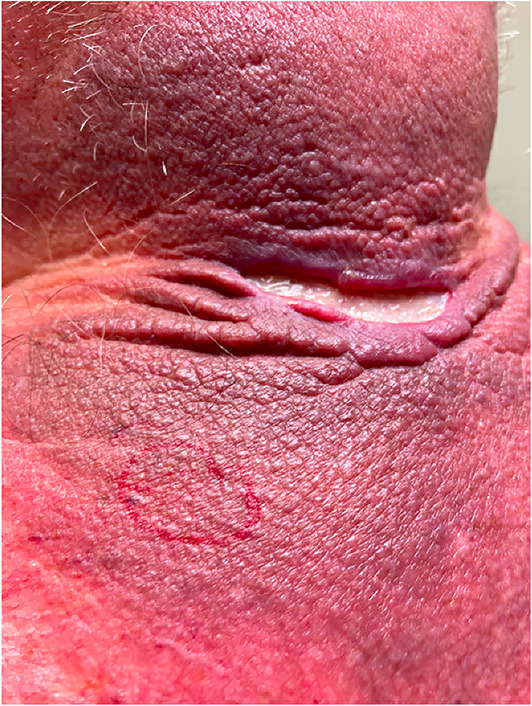

A 65‐year‐old diabetic man, with a history of recent weight gain post‐knee surgery, presents with a worsening year‐long indurated plaque on his back and neck with ulceration (Figures 1 and 2). In addition to severe discomfort, he also had a restricted range of motion in the back and neck. Initial antibiotic treatment for suspected cellulitis proved ineffective, prompting a dermatology referral. At initial presentation, the haemoglobin A1C (11.3%) and blood glucose (282 mg/dL) indicated poor metabolic control. Cases of severe scleredema diabeticorum (SD) may cause skin fissures and secondary infection and obtaining adequate tissue for biopsy is essential for diagnosis (Figure 3). Scleredema of Buschke, also known as SD or scleredema adultorum, is subclassified into three types. Type 1 is acute and typically arises from infection, Type 2 is chronic and arises in instances of paraproteinemia or blood cell dyscrasia, and Type 3, ‘SD’, may present acutely or chronically and is associated with metabolic dysfunction, most commonly type 2 diabetes.^1^ SD is thought to be caused by glycosylation of collagen leading to excessive accumulation in the dermis.^2^ Early lesions of SD are characterized by skin thickening and erythema which later progress to a reddish brown induration with loss of skin wrinkling. Affected areas cause patients discomfort through severe recalcitrant itching or burning sensations.

**FIGURE 1:**
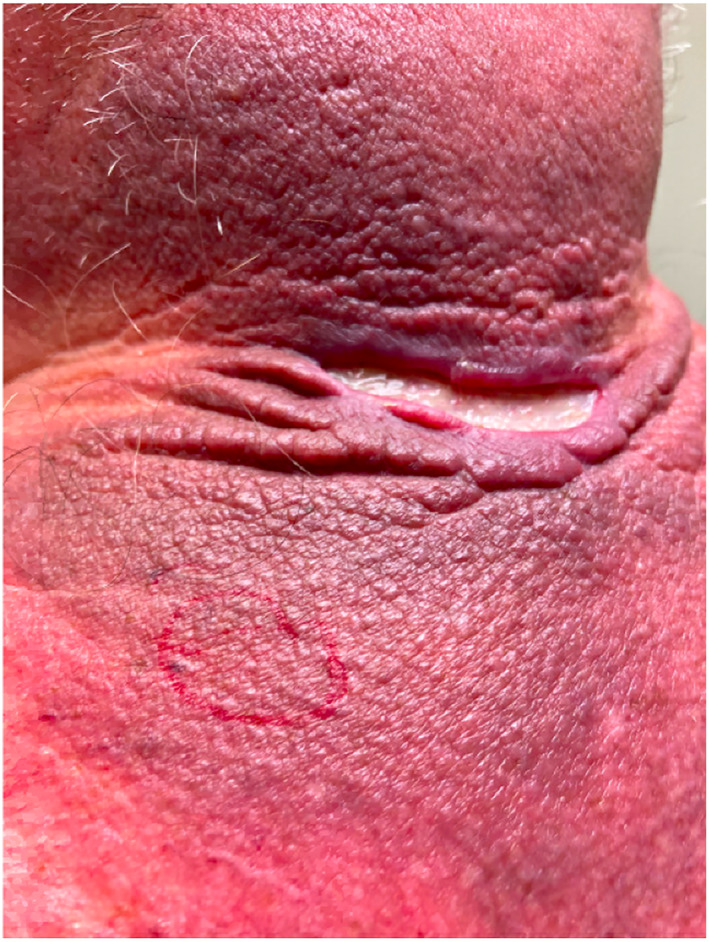
Clinical photograph of the indurated plaque with fissuring of the skin folds.

**FIGURE 2:**
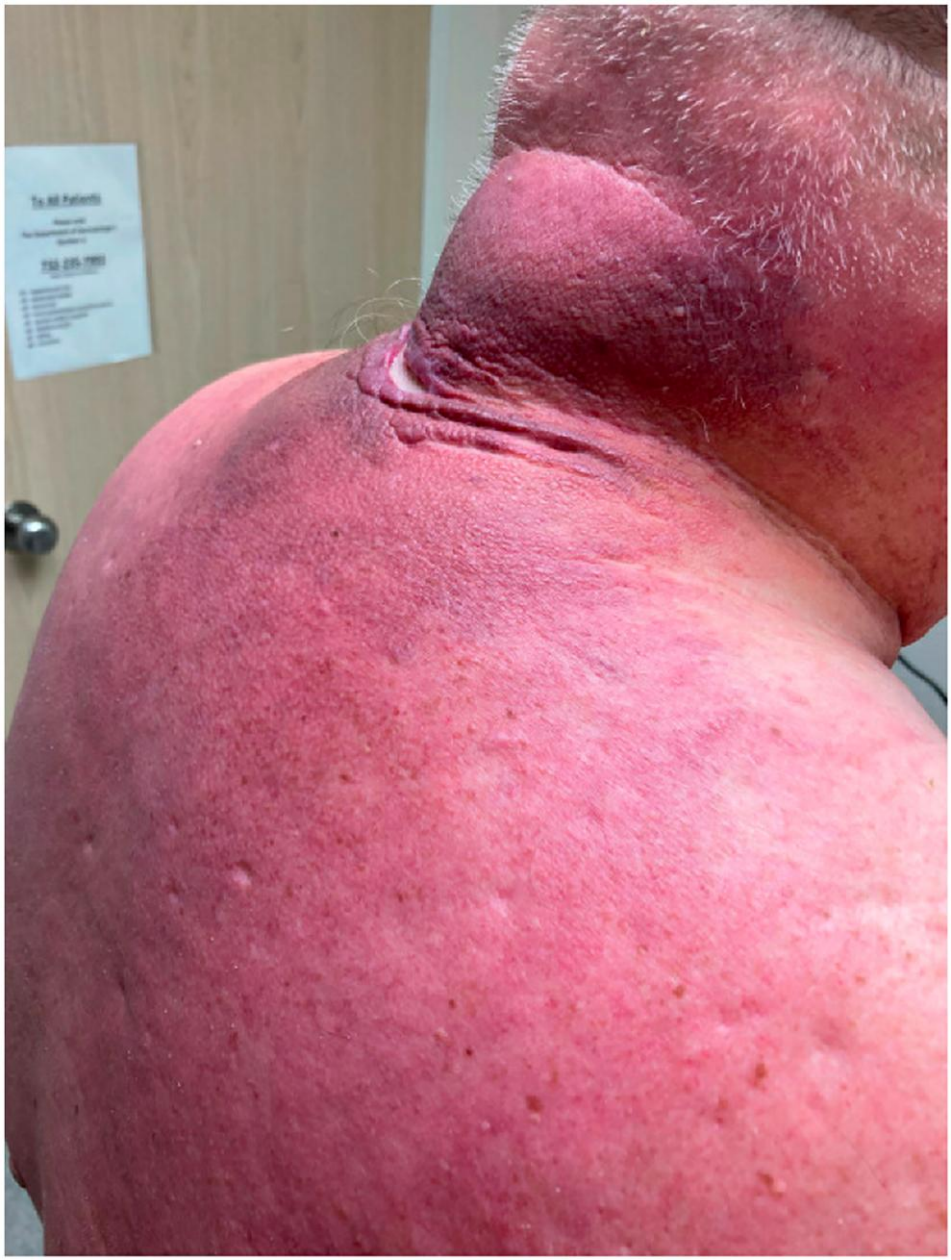
Additional photo demonstrating the extent of the disease.

**FIGURE 3:**
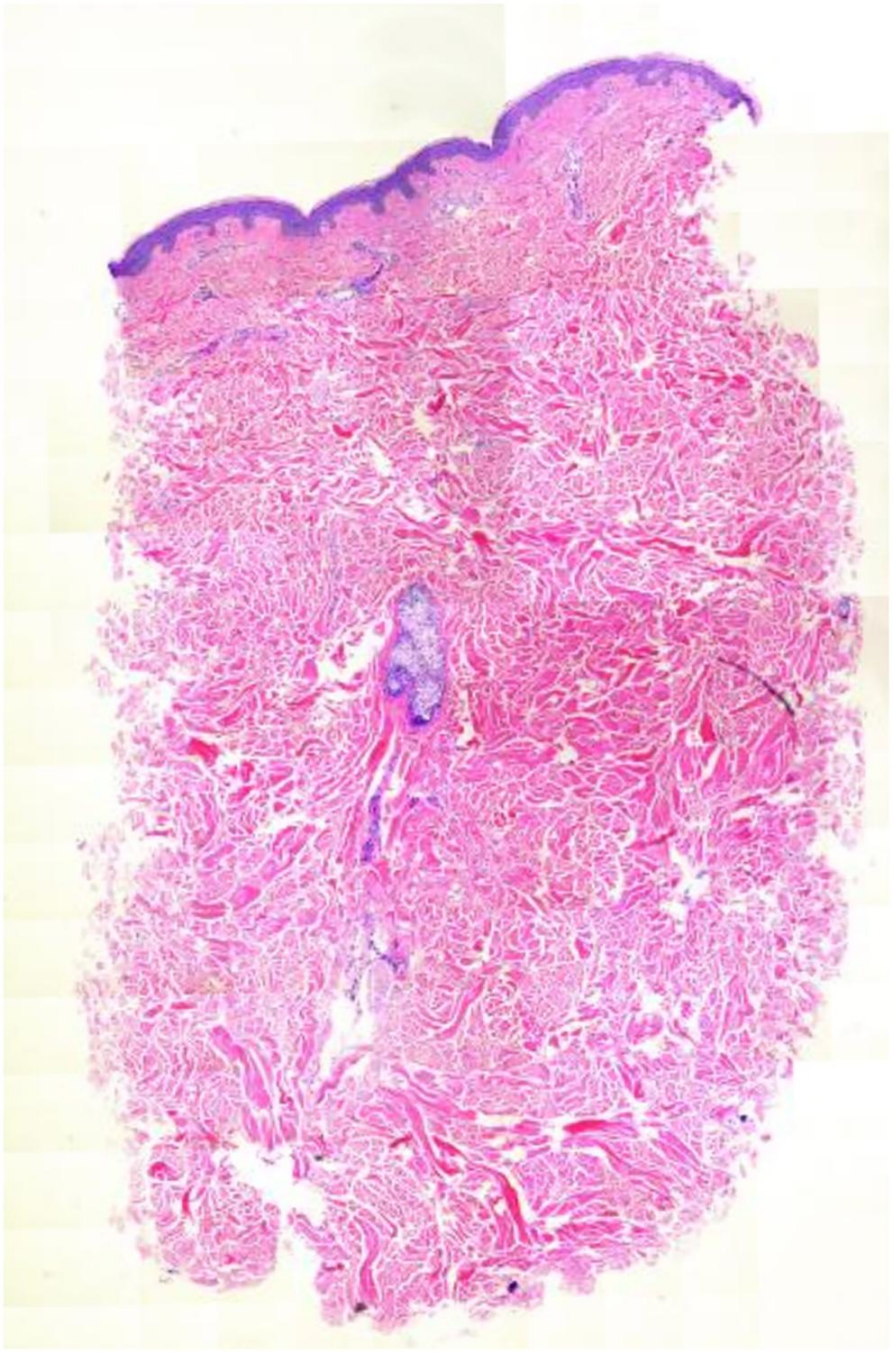
H&E stain, 20× magnification. Notable for an increase in spaces between collagen bundles in the dermis and sparse superficial perivascular dermatitis.

## CONFLICT OF INTEREST STATEMENT

The authors declare no conflicts of interest.

## AUTHOR CONTRIBUTIONS

**Thu M. Truong**: Writing – original draft (lead); writing – review & editing (lead). **Timothy Makkar**: Conceptualization (equal); supervision (equal); writing – original draft (equal); writing – review & editing (equal). **Amy Pappert**: Conceptualization (equal); supervision (equal); writing – original draft (equal); writing – review & editing (equal).

## ETHICS STATEMENT

This study is exempt from IRB review and appropriate consent has been obtained for studies involving human participants.

## Data Availability

The data underlying this article will be shared on reasonable request to the corresponding author.
